# Marine Antitumor Peptide Dolastatin 10: Biological Activity, Structural Modification and Synthetic Chemistry

**DOI:** 10.3390/md19070363

**Published:** 2021-06-24

**Authors:** Gang Gao, Yanbing Wang, Huiming Hua, Dahong Li, Chunlan Tang

**Affiliations:** 1 School of Medicine, Ningbo University, 818 Fenghua Road, Ningbo 315211, China; gaogang9213@163.com; 2 Key Laboratory of Structure-Based Drug Design & Discovery, Ministry of Education, and School of Traditional Chinese Materia Medica, Shenyang Pharmaceutical University, 103 Wenhua Road, Shenyang 110016, China; huimhua@163.com (H.H.); lidahong0203@163.com (D.L.); 3 School of Life Science and Biopharmaceutics, Shenyang Pharmaceutical University, 103 Wenhua Road, Shenyang 110016, China; wangyanbing96@163.com

**Keywords:** marine peptide, dolastatin 10, antitumor, lead exploration

## Abstract

Dolastatin 10 (Dol-10), a leading marine pentapeptide isolated from the Indian Ocean mollusk *Dolabella auricularia*, contains three unique amino acid residues. Dol-10 can effectively induce apoptosis of lung cancer cells and other tumor cells at nanomolar concentration, and it has been developed into commercial drugs for treating some specific lymphomas, so it has received wide attention in recent years. In vitro experiments showed that Dol-10 and its derivatives were highly lethal to common tumor cells, such as L1210 leukemia cells (IC_50_ = 0.03 nM), small cell lung cancer NCI-H69 cells (IC_50_ = 0.059 nM), and human prostate cancer DU-145 cells (IC_50_ = 0.5 nM), etc. With the rise of antibody-drug conjugates (ADCs), milestone progress was made in clinical research based on Dol-10. A variety of ADCs constructed by combining MMAE or MMAF (Dol-10 derivatives) with a specific antibody not only ensured the antitumor activity of the drugs themself but also improved their tumor targeting and reduced the systemic toxicity. They are currently undergoing clinical trials or have been approved for marketing, such as Adcetris^®^, which had been approved for the treatment of anaplastic large T-cell systemic malignant lymphoma and Hodgkin lymphoma. Dol-10, as one of the most medically valuable natural compounds discovered up to now, has brought unprecedented hope for tumor treatment. It is particularly noteworthy that, by modifying the chemical structure of Dol-10 and combining with the application of ADCs technology, Dol-10 as a new drug candidate still has great potential for development. In this review, the biological activity and chemical work of Dol-10 in the advance of antitumor drugs in the last 35 years will be summarized, which will provide the support for pharmaceutical researchers interested in leading exploration of antitumor marine peptides.

## 1. Introduction

The living environment of marine organisms is different from that of terrestrial creatures, and the peptide secondary metabolites of the former have special structures and provide bioactive components distinct from the latter [1,2,3]. Since the beginning of the 21st century, the potential of unique marine natural products as candidate drugs has been widely recognized, and significant advancement has been seen in the clinical research of marine-derived medicines [4,5,6,7,8,9,10,11,12,13]. After decades of exploration, a series of antitumor polypeptides with high biological activity have been isolated from various marine organisms, such as hemiasterlins, cryptophycins, vitilevuamide, diazonamide, etc. [14,15]. Structures of these polypeptides are dissimilar from that of terrestrial plant peptides (mainly glycopeptides). Marine peptides are rich in hydroxy acids, thiophenols, and D- and *α*-amino acids. Some components possess ethylenic bonds and acetylenic bonds, which improve the stability and bioavailability of peptides [16,17,18,19,20,21]. Compared with traditional small molecule inhibitors, marine peptides present high activity, strong targeting, low toxicity, easy transmembrane absorption, and own a wide range of biological activities, such as antitumor, anti-inflammatory, antivirus, and antiinfection. Currently, an increasing number of candidate drugs derived from marine peptides have entered clinical trials or been approved for marketing [22,23,24,25,26,27,28].

**Figure 1:**
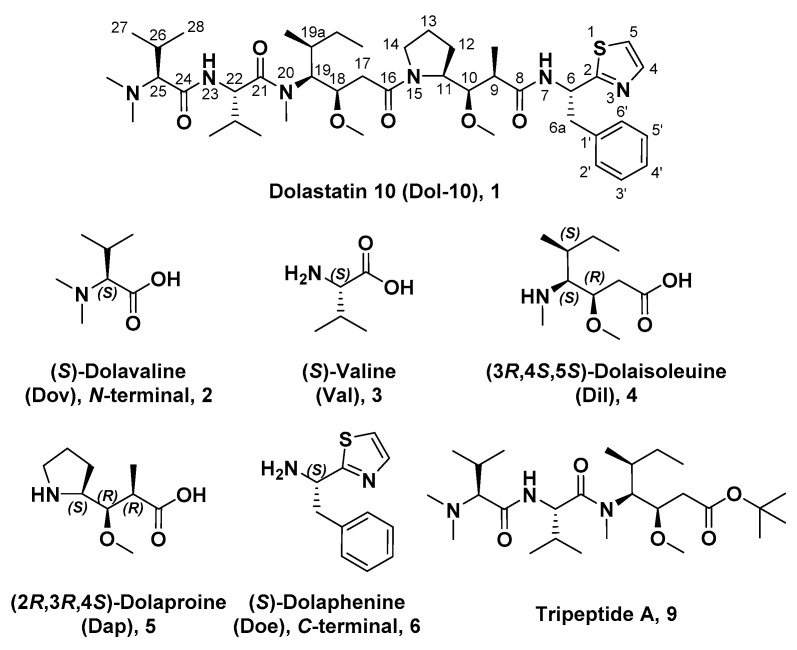
Chemical structures of Dol-10 (**1**), key amino acid components (**2–6**) of Dol-10 were ordered from *N*-terminal to *C*-terminal and tripeptide A (**9**).

The bioactive antitumor components from mollusk *Dolabella auricularia*, a sea hare that mainly inhabits the Indian Ocean, were first investigated by Pettit and his co-workers. Since 1972, they had gradually discovered 18 short-chain peptide compounds, named as dolastatins 1–18, which contained several unusual amino acids. In the dolastatins family, dolastatin 10 (Dol-10, **1**, Figure 1) had been proved to be one of the most potent antiproliferative active ingredients, and it allowed for prolonging the life span of P388 leukemia mice [21,29,30,31]. The chemical structures of its constituent subunits were analyzed, and all four subunits, starting from the amino *N*-terminal of Dol-10, (*S*)-dolavaline (Dov, **2**, Figure 1), (*S*)-valine (Val, **3**), (3*R*,4*S*,5*S*)-dolaisoleuine (Dil, **4**), and (2*R*,3*R*,4*S*)-dolaproine (Dap, **5**), were actual amino acid residues, while (*S*)-dolaphenine (Doe, **6**), a peculiar primary amine possibly stemmed from phenylalanine, worked as carboxyl *C*-terminal. Even so, researchers were still accustomed to regard Dol-10 as a pentapeptide [32,33,34].

**Figure 2:**
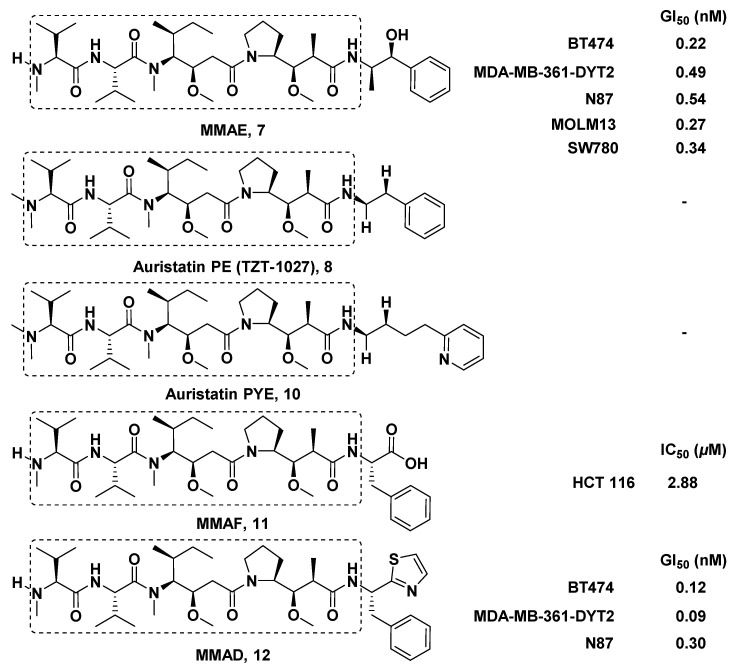
Chemical structures of key auristatin analogs.

Excitedly, since Dol-10 was first discovered, its biological activity was more potent than those of most known anticancer drugs (murine PS leukemia cells, ED_50_ = 4.6 × 10^−5^ μg/mL), so it had attracted extensive attention in the field of anticancer research. In in vitro antitumor screening, it was found that Dol-10 had a strong antiproliferation effect on L1210 leukemia cells (IC_50_ = 0.03 nM) [35], non-Hodgkin lymphoma cells [36], B16 melanoma cells, small cell lung cancer NCI-H69 cells (IC_50_ = 0.059 nM) [37], human prostate cancer DU-145 cells (IC_50_ = 0.5 nM) [38], etc. In addition, it had been confirmed that the ability of Dol-10 to induce apoptosis mainly came from its effective inhibition of tubulin polymerization [30,35,39,40,41]. However, the rare content in organisms brings great obstacles to the enrichment and further biological evaluation of bioactive peptides. Because of the relatively simple chemical structure, scientists prepared a large amount of Dol-10 by chemical synthesis, which laid the foundation for its further development and clinical evaluation [16,21,42]. Due to its adverse effects, such as the most common peripheral neuropathy, Dol-10 was stagnated in phase II clinical trial [43,44]. It is inspiring to know that a lot of synthetic analogs of Dol-10 with potent antitumor activity have been reported. For example, TZT-1027 (Soblidotin) entered the phase I clinical trial, the main toxicity and neutropenia were observed, and the dosage level was insufficient to achieve clinical efficacy [45,46]. MMAE (a Dol-10 derivative, **7**, Figure 2) conjugated with the special monoclonal antibody to construct the Adcetris^®^ (Brentuximab vedotin), and Adcetris^®^ was approved by the FDA in 2011 for the treatment of anaplastic large T-cell systemic malignant lymphoma and Hodgkin lymphoma [47].

Up to now, a large number of research articles have reported on Dol-10 and its derivatives, but to the best of our knowledge, there are few literatures that systematically summarized Dol-10. Therefore, this review will introduce the pharmacological characteristics of Dol-10, the antitumor activity of its structurally modified derivatives, chemical synthesis of Dol-10 and its subunits, and its development and application on the basis of ADCs in detail.

## 2. Pharmacological Characteristics

The in vitro evaluation of pharmacological activity showed that Dol-10 could cause apoptosis of many tumor cells at nanomolar concentrations, the strong antitumor activity came from the unique mechanism of inducing apoptosis, including interactions with *β*-tubulin, regulating the expression of special oncoproteins, loss of telomeric repeat sequence of chromosome and provoking chromosome aberrations. In the application of Dol-10 in various tumor treatment (Table 1), the therapeutic activity in clinical trials was obviously inferior to that, in preclinical trials, and the reason remained uncertain. Since accumulated retention in cells and high affinity binding with tubulin might affect the therapeutic effect of Dol-10, it was speculated that the administration scheme would interfere with the clinical evaluation results, and infusion administration was superior to bolus administration [48,49]. Furthermore, to provide reasonable guidance for clinical application, and ensure the safety and effectiveness of chemotherapy, the pharmacokinetics, pharmacodynamics, toxicity, and drug resistance of Dol-10 had also been explored in preclinical and phase I clinical trial. All of the above investigations will be elaborated through the following sections.

### 2.1. Antitumor Effects

#### 2.1.1. Anti-Lymphoma Effect

Lymphoma is a malignant tumor originated from the lymphohematopoietic system, and it is also a disease that threatens human life. Fortunately, Dol-10 had significant antiproliferative activity in four diverse human lymphoma cell lines (DB, HT, RL, and SR), which was about 3–4 logarithms stronger than vincristine. The inhibitory effect of tumor cells could be reversed by removing the agent within the first 4 h of treatment [50]. Maki et al. confirmed that Dol-10 would effectively trigger the apoptosis of non-Hodgkin lymphoma cells [36]. In the model of human diffuse large cell lymphoma cell line (WSU-DLCL 2), the antitumor activity of Dol-10 was not obvious at concentrations less than 500 pg/mL, but auristatin PE (TZT-1027, **8**, a typical derivative of Dol-10, Figure 2) possessed the remarkable antiproliferative ability at concentrations as low as 10 pg/mL, and the administration concentration was allowed to be 10-fold higher than that of Dol-10 [51]. The uptake and efflux of Dol-10 and vinblastine in human Burkitt lymphoma CA46 cells were studied by radioactive labeling. It was found that the tight binding of Dol-10 with tubulin not only prolonged intracellular residence time, but also determined the preferential activity. In addition, in clinical application, the best way to administer Dol-10 might be infusion rather than bolus injection [49].

#### 2.1.2. Anti-Small Cell Lung Cancer Effect

Overexpression of Bcl-2 is common in small cell lung cancer (SCLC), and Dol-10 has the ability of inducing Bcl-2 phosphorylation to induce apoptosis in SCLC cell lines and xenografts. In the related efficacy evaluation, the standardized MTT assay was employed to estimate the growth inhibitory efficiency in vitro, and the in vivo activity was evaluated based on SCID mice subcutaneous and metastatic SCLC xenoplantation models. The results showed that the growth of four SCLC cell lines (NCI-H69, NCI-H82, NCI-H446, and NCI-H510) was strongly inhibited through Bcl-2 phosphorylation triggered by Dol-10 (IC_50_ = 0.032–0.184 nM) [37].

#### 2.1.3. Anti-Ovarian Carcinoma Effect

In order to evaluate the therapeutic efficacy of Dol-10 on relapsed platinum-sensitive ovarian carcinoma, the patients were injected with 400 μg/m^2^ of Dol-10 intravenously every three weeks, and the tumor status was measured 1–2 cycles after administration. Under the limited level of toxicity, the drug was only effective for some patients, which indicated that Dol-10 needed further improvement for the treatment of platinum-sensitive recurrent ovarian cancer [48].

#### 2.1.4. Anti-Prostate Cancer Effect

In the tissue culture and athymic nude mice experiment, the growth of human prostate cancer DU-145 cells was completely inhibited by 1 nM Dol-10 (IC_50_ = 0.5 nM) [36]. In the phase II clinical trial of patients with hormone refractory prostate cancer, Dol-10 showed excellent tolerance, especially among the elderly pre-treatment population, but the clinical efficiency as a single drug was seriously insufficient [52]. 

#### 2.1.5. Anti-Soft Tissue Sarcoma Effect

In the phase II trials of Dol-10, the therapeutic activity of this microtubule inhibitor to recurrent or metastatic soft tissue sarcoma was verified by Mehren et al. During the course of treatment, no patient got obvious improvement, but one died of respiratory failure, which indicated that Dol-10 was not warranted for further research on the chemotherapy for advanced or metastatic soft tissue sarcoma [53].

#### 2.1.6. Anti-Breast Cancer Effect

Dol-10 was also applied as the first-line or second-line chemotherapy for advanced breast cancer, and the results showed that the marine polypeptide had no significant activity. In addition, the drug was found to have hematological toxicity in an acceptable range. It was speculated that a large dose of Dol-10 might overcome its drug resistance and induce effective antitumor activity [54]. 

#### 2.1.7. Anti-Hepatobiliary Pancreatic Carcinoma Effect

With the aim of characterizing the efficacy and toxicity of Dol-10 for patients with advanced hepatobiliary cancer and pancreatic cancer, Kindler et al. performed two parallel phase II trials. Patients with metastatic pancreatic cancer, liver cancer, cholangiocarcinoma, or gallbladder cancer and had not received corresponding chemotherapy before, were eligible for the trials. The results showed that the toxicity of Dol-10 was tolerable, but there was no obvious therapeutic effect [55].

### 2.2. Mechanisms of Inducing Apoptosis

#### 2.2.1. Interactions with β-tubulin

**Figure 3:**
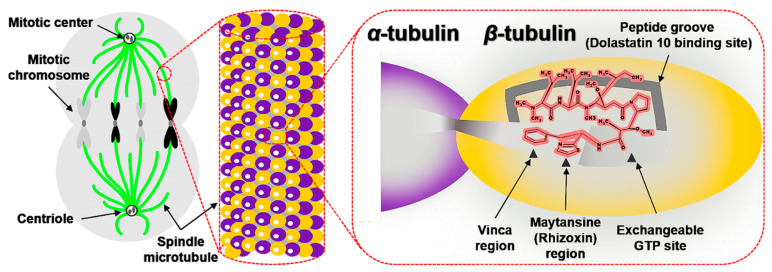
The graphical model of spindle microtubules in the mitosis and binding relationship of between ligands and *β*-tubulin.

Spindle microtubule was one of the key proteins affecting cell proliferation, which was composed of *α*- and *β*-tubulin (Figure 3). At the late stage of cells division, spindle microtubules pulled sister chromatids from the equatorial plane to the poles of the spindle. After mitosis, spindle microtubules were depolymerized [32,35,41]. Hence, if the equilibrium (polymerization and depolymerization) of tubulin-microtubule is broken, the mitosis would stagnate in the G_2_/M phase, which further inhibits the growth or induces apoptosis of tumor cells [56,57,58,59,60].

Dol-10, interfered with tubulin-microtubule balance, had potential to be developed as a new antimitotic drug. It was generally believed that the binding sites (more than one) of Dol-10 (Figure 3) located in the *β*-tubulin subunits in the central part of spindle microtubules [58,61,62], and there was no interaction between *α*-tubulin and Dol-10 [63]. The combination of Dol-10 and *β*-tubulin would trigger a series of activities related to apoptosis. For example, microtubule assembly, tubulin-dependent GTP hydrolysis and *β*^s^ cross-linking were all inhibited, but microtubule depolymerization was enhanced [33,64]. In addition, a schematic model of the relationship between binding sites of Dol-10, vinca, maytansine/rhizoxin, and exchangeable GTP site on *β*-tubulin was proposed (Figure 3). Tripeptide A (**4**, Figure 1) was a tripeptide residue at the *N*-terminal of Dol-10 and located in a hydrophobic pocket or groove which was part of the binding site of Dol-10 but had little or no overlap with the other three binding sites. Therefore, it was speculated that the other two residues at the *C*-terminal of the Dol-10 would hinder the binding of vinca alkaloids and GTP exchange in space. The accuracy and rationality of the above model were confirmed in a series of experiments [65,66,67,68]. As the noncompetitive inhibitor of vinca alkaloids, Dol-10 targeted vinblastine, maytansine/rhizoxin binding domain in *β*-tubulin to realize the microtubule inhibition activity. Later work verified that tripeptide A (**4**) inhibited tubulin polymerization but hardly disturbed the binding of vincristine to tubulin and GTP exchange [33,62,64,69]. In addition, the *β*^s^ cross-link between the sulfur atom of cysteine 12 of *β*-tubulin and thiazole portion of Dol-10 interfered with the binding of Dol-10 to *β*-tubulin. Auristatin PE, the equivalent analog of Dol-10 lacking thiazole ring, failed to establish this cross-link. Model research also confirmed that the binding site of Dol-10 (possibly containing cysteine 12) was adjacent to the exchangeable GTP site [63,70]. 

#### 2.2.2. Regulating the Expression of Bcl-2, c-myc, and p53

**Figure 4:**
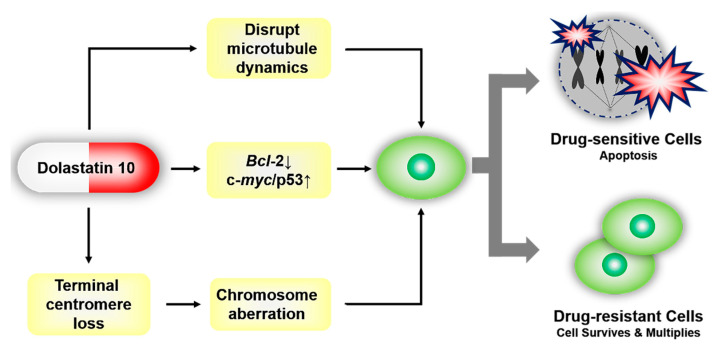
Dol-10-mediated reactivation of the apoptosis pathway in tumor cells. Drug-sensitive tumor cells were arrested at the G2/M phase of the cell cycle, and then apoptosis occurred. Resistance to Dol-10 led to the survival of drug-resistant cells.

Oncoproteins, such as coding products of oncogenes *Bcl*-2, c-*myc*, and tumor suppressor gene p53, are the critical functional proteins in the apoptosis signal pathway. It is significant to further mediate cell proliferation and apoptosis by regulating the expression of oncoproteins. Dol-10 down-regulated or inhibited the expression of anti-apoptotic Bcl-2 protein and promoted the overexpression of c-*myc* or p53, which as the initial signal of reactivating apoptosis pathway would further induce apoptosis of tumor cells (Figure 4) [36,37,71]. Bcl-2 phosphorylation and apoptosis induced by Dol-10 were discussed by Haldar et al. in 1998. In the G_2_/M phase of the cell cycle, it was observed by site-directed mutagenesis that several serine residues (in charge of drug-induced phosphorylation of Bcl-2) on Bcl-2 protein were translated and then induced to phosphorylation by Dol-10, which led to apoptosis of malignant tumor cells [72]. 

#### 2.2.3. The Loss of Telomeric Repeats and Induction of Chromosome Aberrations

In addition to the inhibition of microtubule aggregation and induction of Bcl-2 phosphorylation, the mechanism of inducing apoptosis mediated by loss of telomere repeats sequence and chromosomal aberrations had also aroused researchers’ interest. The efficacy of Dol-10 on chromosome morphology, telomere association, polyploid induction and apoptosis of K1735 clone X-21 were identified based on the mouse metastatic melanoma cell line model. The investigation suggested that the loss of telomere repeats promoted the formation of telomeric associations, multicentric chromosomes and ring configurations, and led to chromosome aberration, which eventually contributed to cell death induced by Dol-10 (Figure 4) [73,74,75]. 

### 2.3. Pharmacokinetics and Pharmacodynamics

From preclinical research to the clinical trial, it was necessary to study the pharmacokinetics and pharmacodynamics of Dol-10 in detail, especially to measure the maximum tolerated dose (MTD), so as to achieve the maximum therapeutic activity, reduce the adverse reactions and ensure that each patient receives the best dose of treatment. An assay based on HPLC/ESI-MS was employed to characterize the clinical pharmacokinetics and pharmacodynamics of Dol-10. Preliminary examination showed that the three-compartment model was the optimum approach to describe the distribution and elimination of Dol-10, and *t*_1/2α_, *t*_1/2β_, and *t*_1/2γ_ were 0.087 h, 0.69 h, and 8.0 h, respectively. At MTD level (about 300 μg/m^2^), 33% of the treated patients with advanced solid tumors had granulocytopenia (the dose-limiting toxicity), but it was well-tolerated, and some patients with stable tumor growth had no objective reaction to the drug. Pharmacokinetic analysis revealed that the drug distribution was rapid, but the process of plasma elimination was relatively slow (with a *t*_1/2z_ of 5.3 h). The change of Dol-10 concentration with time was closely related to the decrease of white blood cell count [44,76,77,78]. Given all that, Dol-10 showed good preclinical characteristics and can be used for further clinical research.

### 2.4. Toxicity

According to the results of preclinical toxicology research, it is of great significance to predict the dose-limiting toxicity of the safe initial dose to human body for clinical guidance. The treatment of Dol-10 for advanced or metastatic soft tissue sarcoma triggered hematological and vascular toxicity, but there was no apparent gastrointestinal, liver or kidney toxicity [53]. The preclinical toxicity evaluation on this cytotoxic peptide had proved that the transient reversible toxicity symptoms were mainly concentrated in bone marrow of ordinary mammals. In this animal experiment, granulocytopenia and bone marrow toxicity were dose-limited, and the Dol-10 MTD of dogs and humans was equivalent (about 325–455 μg/m^2^) [79]. The temporary adverse reactions of Dol-10 to dogs with non-Hodgkin lymphoma included granulocytopenia with dose-limiting toxicity, and this examination predicted that the initial dose of 300 μg/m^2^ was suitable for human beings [80]. Since 1990, Dol-10 had successively entered the phase I and II clinical trials, preliminary statistics presented that 40% of patients helped with Dol-10 had moderate peripheral neuropathy [43]. Some patients accompanied by bone marrow suppression and phlebitis, etc. If the patient had potential mental illness, then the side effects would be more obvious [81].

### 2.5. Drug Resistance

Dol-10 as a new member of multidrug resistance (MDR) phenotype expressed by sublines of murine PC4 and human U-937 leukemia cells, the drug resistance mechanism (Figure 4) and how to ensure the effectiveness and rational application of Dol-10 had attracted widespread concern. In the parallel investigations in a CHO cell line transfected with human *mdr*1 cDNA, Toppmeyer et al. had confirmed the resistance of transfected cells to Dol-10 was originated from the normal expression of P-glycoprotein (P-gp), and this resistance could be reversed by verapamil. The employment of Dol-10 resulted in a decrease in the photoaffinity labeling of P-gp, which further demonstrated the binding of Dol-10 to P-gp. The drug resistance to Dol-10 was at least related to the expression of the *mdr*1 gene and the interaction between Dol-10 and P-gp [82].

## 3. Medicinal Chemistry

Due to the unexpected results of Dol-10 in phase II clinical trials, researchers turned their attention to its antitumor derivatives. These derivatives mainly focused on *N*-terminal (Dov) and *C*-terminal (Doe) subunits with great potential for drug development. However, there were relatively fewer modifications of central residues Dil, Dap, and Doe [83,84]. Auristatins (Figure 2) based on the bipolar modification of Dol-10 had potent microtubule inhibitory activity and cytotoxicity. Auristatin PE (TZT-1027) and auristatin PYE (**10**, Figure 2) showed remarkable therapeutic effects in clinic. However, due to the complicated chemical structure and low water solubility, the clinical research progress of these drugs was not satisfactory. Later, with the rise of research on ADCs, the application of auristatins as payload provided an excellent platform for the preparation of ADCs. Related ADCs, such as Adcetris^®^, which successfully went on the market, also made notable progress in clinical trials [85]. Design, synthesis, and structure-activity relationship of various antitumor derivatives classified according to specific modified subunits are detailed as follows. 

### 3.1. Antitumor Derivatives

#### 3.1.1. Doe Unit Modified Derivatives

By replacing Doe residue with Met, Phe, or appropriately modified phenethylamide, the activity of synthesized derivatives in interfering with microtubule assembly, vinblastine, and GTP targeting tubulin and mitosis of various human and mouse cancer cell lines was evaluated. Moreover, it was confirmed that the antitumor ability of some Dol-10 derivatives was equivalent to that of Dol-10 [86]. Auristatin PYE, derived from Doe unit of Dol-10 replaced by pyridine group, interfered with polymerization and stability of microtubules in micromole concentration range, leading to apoptosis and growth inhibition of tumor cells [87]. Auristatin PE (TZT-1027), a Dol-10 derivative synthesized by replacing Doe unit with phenylethylamine, had the same mechanism as Auristatin PYE, expanded the antitumor spectrum of the pentapeptide [88], and exhibited higher in vivo antitumor activity than other anticancer agents, such as paclitaxel, adriamycin, and vincristine, against murine fibrosarcoma Meth A [89,90]. 

**Scheme 1:**
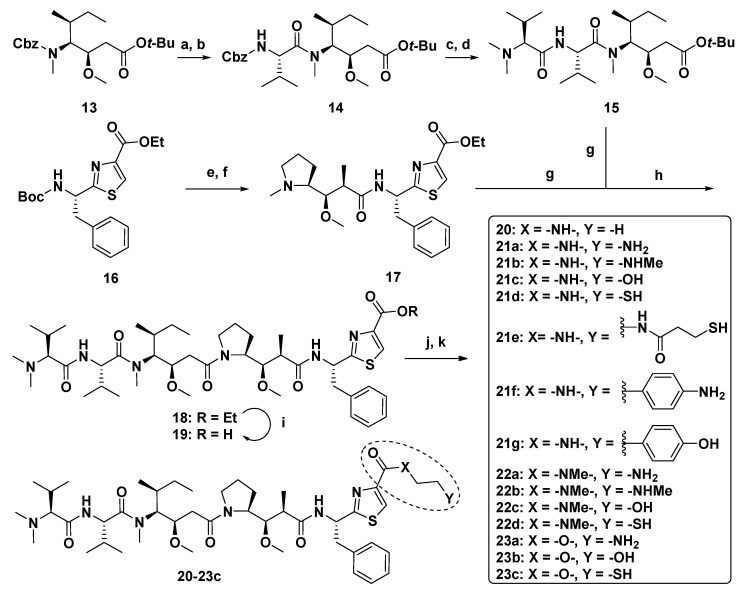
Synthesis of Dol-10 derivatives with *C*-terminal modifications. Reagents and conditions: (a) H_2_, Pd/C, MeOH, 71%; (b) Cbz-Val-OH, COMU, DIPEA, DMF, 97%; (c) H_2_, Pd/C, MeOH, 93%, (d) Dov-OH, HATU, DIPEA, DMF, 75%; (e) HCl, AcOEt, 99%; (f) Boc-Dap-OH, HATU, DIPEA, DMF, 85%; (g) TFA, CH_2_Cl_2_; (h) HATU, DIPEA, DMF, 83%; (i) NaOH, EtOH, quant; (j) amine, HATU, DIPEA, DMF, 43–100% (**17**, **21a**–**g**, **22a**–**d**, and **23a**) or alcohol, 2-methyl-6-nitrobenzoic anhydride, Et_3_N, DMAP, CH_2_Cl_2_, 73–92% (**23b**–**c**); (k) deprotection of protecting groups (Boc: **21a**–**b**, **22a**–**b**, **23a**, THP: **23b**, Trt: **21d**–**e**, **22d**, **23c**) if needed.

Substituting various functional groups for Doe unit of novel derivatives is also essential to expand the application of Dol-10 analogs in conjugated drugs. A series of Dol-10 derivatives which added a carbonyl functional group to the C-4 of thiazole of Doe unit were synthesized by Yokosaka and co-workers (Scheme 1). In the tumor cell proliferation evaluation, amine, aniline, alcohol, phenol, and thiol derivatives were observed to have extremely strong activity (data shown in Table 2), which were also considered as the versatile payloads of conjugated drugs [91].

**Scheme 2:**
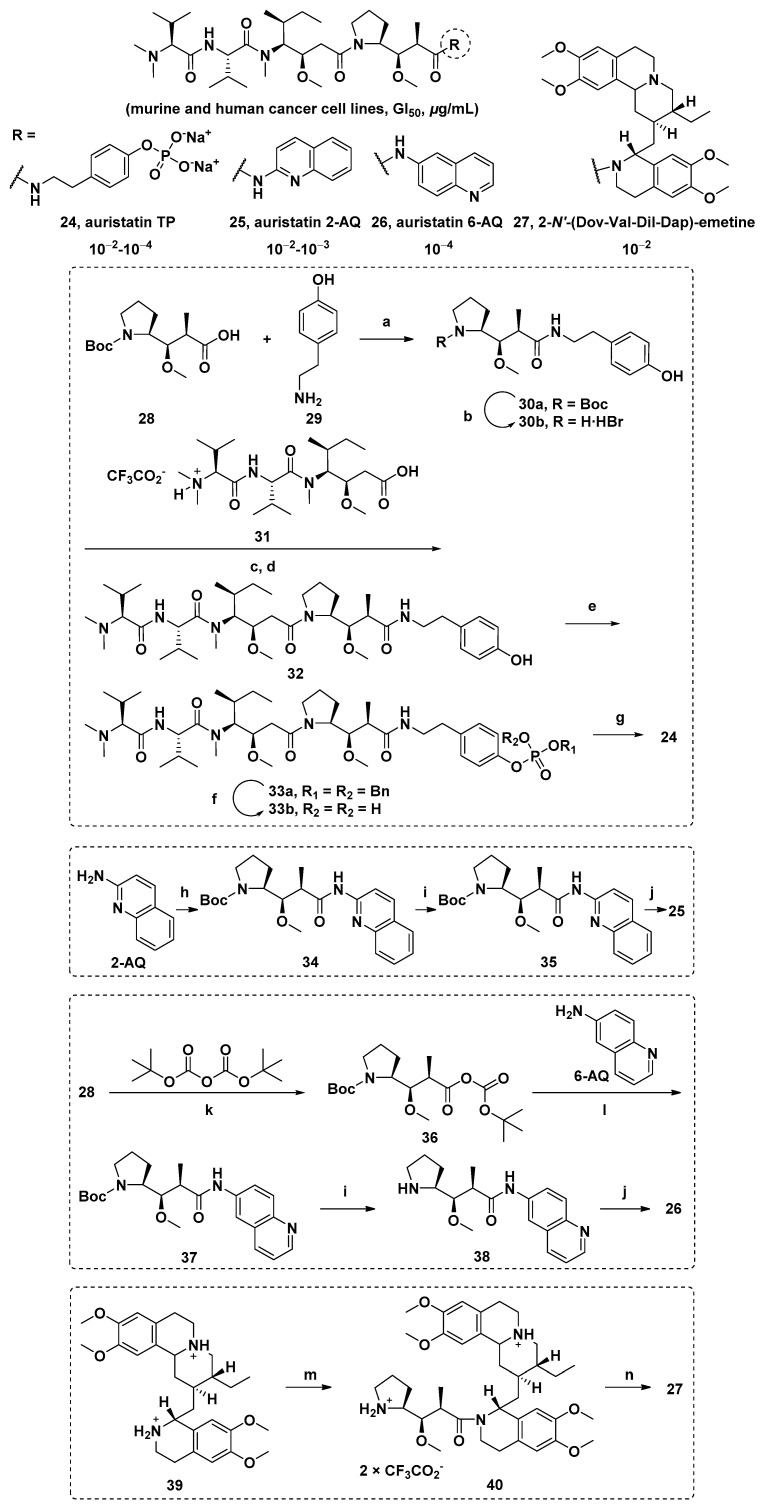
The chemical structures and synthesis of **24**, **25**, **26**, and **27**. Reagents and conditions: (a) (1) EDCI, HOBT; (2) DIEA, DMF; (b) TMSBr; (c) EDCI, HOBT; (d) DIEA, DMF; (e) (BnO)_2_P(O)H, CCl_4_, DIEA, DMAP; (f) Pd/C, H_2_; (g) ion exchange; (h) **28**, DEPC, TEA; (i) TEA; (j) **32**, DEPC, TEA; (k) pyridine; (l) 6-AQ, DEPC, TEA; (m) (1) **28**, TEA, HBTU, DMF; (2)TFA, CHCl_2_; (n) **31**, CH_2_Cl_2_, DEPC, TEA, 0 °C-r.t.

Dol-10 derivatives based on substitution of the Doe unit with phosphate, aminoquinoline (AQ) auristatin isomer 2/6-AQ, and emetine were synthesized. The activity of all new auristatins in mouse and human cancer cell lines was evaluated. The results showed that the sodium phosphate derivative so auristatin TP (**24**), auristatin 2-AQ (**25**), auristatin 6-AQ (**26**), and 2-*N′*-(Dov-Val-Dil-Dap)-emetine (**27**) had remarkable growth inhibitory effects (Scheme 2, Table 3) [92,93].

#### 3.1.2. Dap Unit Modified Derivatives

**Figure 5:**
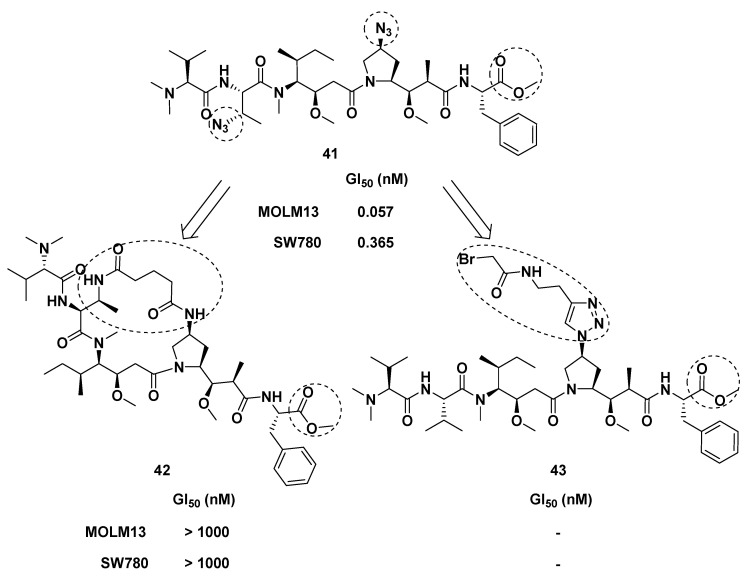
The design and chemical structures of Dol-10 analog (**41**), macrocyclic dolastatin analog (**42**), and drug linker (**43**).

The introduction of azide functional group into Dap subunit enhanced the cytotoxicity of Dol-10 in vitro, which was observed by Akaiwa et al. Inspired by this modification, azide functional groups were introduced into Val subunit and Dap subunit simultaneously, and Doe subunit were replaced by phenylalanine, thus obtaining Dol-10 analog **41** (Figure 5). Compared with MMAE (**7**), the cytotoxic activity of the analog **41** was more potent (GI_50_ = 0.057 nM). These two azide groups not only had the function of regulating growth inhibition but also served as a connecting link to synthesize the brand-new macrocyclic dolastatin analog (**42**) and as a site of drug linker (**43**) attachment for preparing ADCs. Nevertheless, the activity of the synthesized macrocyclic analog (**42**) was disappointingly weak [83]. 

#### 3.1.3. Dil Unit Modified Derivatives

**Figure 6:**
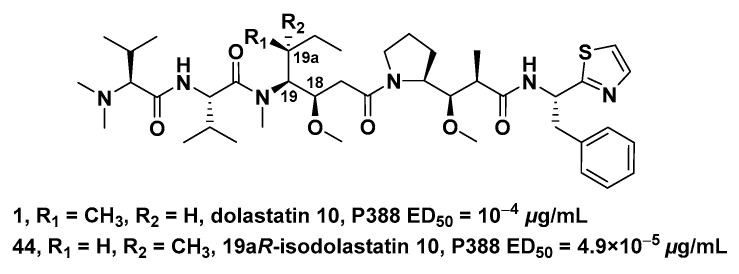
Comparison of chemical structures and activity between (19a*R*)-isodalastatin 10 (**44**) and Dol-10 (**1**).

In the cytotoxicity evaluation of P388 lymphocytic leukemia (PS system), (19a*R*)-isodalastatin 10 (the chiral isomer of Dil unit, **44**, ED_50_ = 4.9 × 10^−5^ μg/mL, Figure 6) had 10-fold stronger inhibitory activity on cell growth than Dol-10 (**1**, ED_50_ = 10^−4^ μg/mL). It had been proved that the antitumor effect was extremely sensitive to the stereochemical modification of the Dil unit (C-18 and C-19). In addition, the general reaction sequence for the synthesis of Dol-10 was also suitable for the total synthesis of 19a*R*-Dil sequence products (including **44**) [94]. Nie et al. established a diastereoselective method to prepare Dil modified derivatives by SmI_2_-induced radical addition reaction. These crucial fragments were applied in the divergent synthesis of Dol-10 (**1**), and nine of the analogs of Dol-10 were obtained. It is worth expecting that, if the antitumor activity of these analogs could be evaluated through relevant biological experiments, it would be helpful for the further development of this synthetic strategy in this field [95].

**Figure 7:**
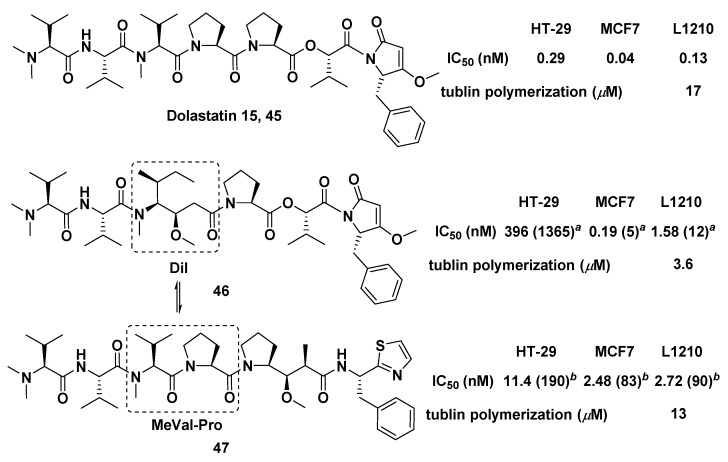
Chemical structures of dolastatin 15 (**45**), **46**, and **47**. Inhibition of cell growth (IC_50_, nM) of Dol-10: HT-29 (0.06), MCF7 (0.03), and L1210 (0.03); tubulin polymerization of Dol-10 (IC_50_ = 2.2 μM). ^a^ Ratio IC_50_ (**46**)/IC_50_ (dolastatin 15); ^b^ Ratio IC_50_ (**47**)/IC_50_ (Dol-10).

Both marine peptides Dol-10 and dolastatin 15 (**45**, Figure 7) had an inextricable connection in structure or biological activity spectrum. The synthesis of compounds **46** and **47** was conducted by replacing the MeVal-Pro dipeptide in dolastatin 15 with the central Dil residue of Dol-10, and vice versa. It was confirmed that both Dil unit and MeVal-Pro dipeptide were essential to cytotoxicity of dolastatins, but the former was superior to the latter in terms of cytotoxicity and inhibition of tubulin polymerization in vitro [96].

#### 3.1.4. Val Unit Modified Derivatives

**Figure 8:**
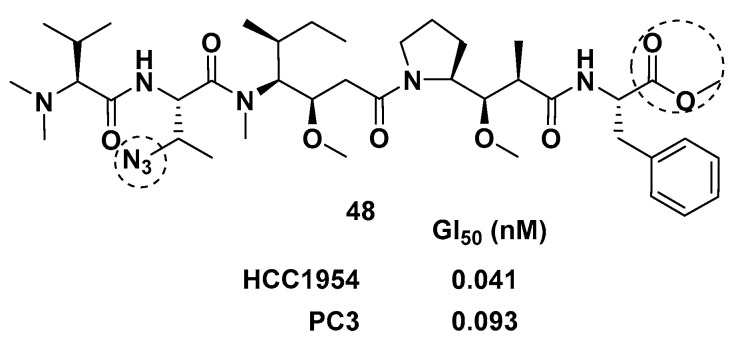
The chemical structure of Val unit modified derivative **48**.

By introducing heteroatoms to the Val unit, Tessier and co-workers obtained a series of auristatins. Dol-10 analog **48** (Figure 8) with Val and Doe subunits modified by azide and phenylalanine, respectively, had effective cytotoxicity when applied to ADCs. Through activity analysis, it was found that phenylalanine replacing Doe unit was crucial in enhancing the in vitro activity of Val unit modified derivatives. Further work confirmed that the modification of the Val unit also needed to modify the Doe unit into an aromatic structure containing ester or amide [84].

#### 3.1.5. Dov Unit Modified Derivatives

**Scheme 3:**
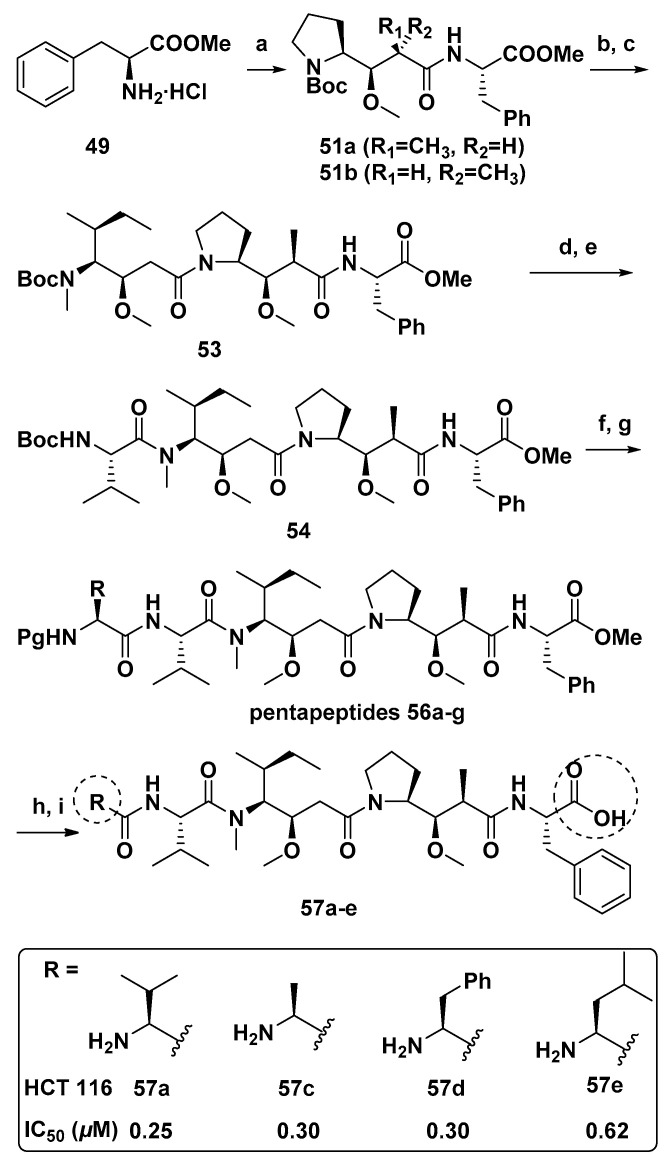
Synthesis of compounds **57a**–**e**. Reagents and conditions: (a) DEPC, DIPEA, DMF, **50a** or **50b** (83–90%); (b) TFA, CH_2_Cl_2_; (c) DEPC, DIPEA, DMF, **52a** or **52b** (76–85%); (d) TFA, CH_2_Cl_2_; (e) Brop, DIPEA, CH_2_Cl_2_, *N*-Boc-*L*-valine, (71–92%); (f) TFA, CH_2_Cl_2_; (g) DEPC, DIPEA, DMF, **55a–g** (please refer to Reference [98]), (46–87%); (h) LiOH·H_2_O, MeOH/H_2_O; (i) TFA, CH_2_Cl_2_ or Pd/C, H_2_, MeOH, 82–94%.

Four Dol-10 analogs of monomethyl auristatin F (MMAF, **11**, Figure 2) modified by *N*-terminal (Dov unit) were designed and synthesized (Scheme 3). These four analogs were more cytotoxic than MMAF against HCT 116 human colon cancer cells [97].

**Scheme 4:**
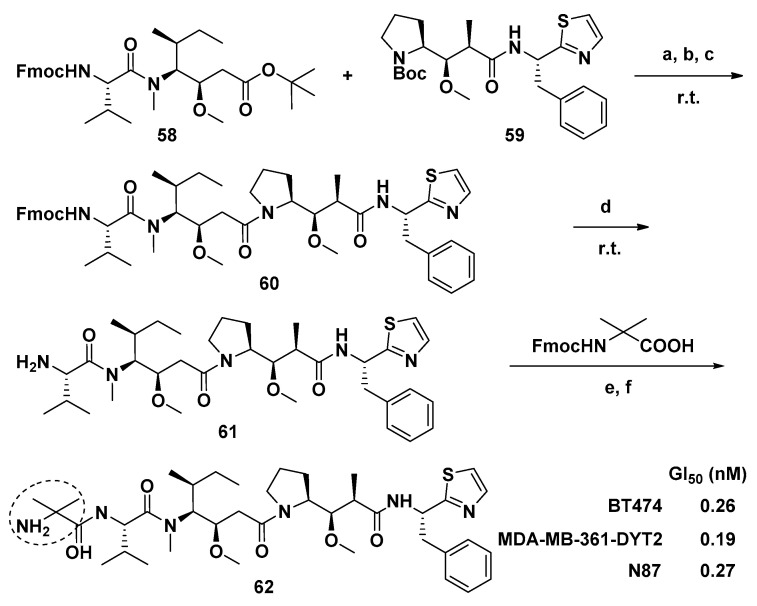
Synthesis of new auristatin analog **62**. Reagents and conditions: (a) TFA/DCM for **58**; (b) HCl dioxane for **59**; (c) HATU, *i*-Pr_2_NEt, DCM, 78%; (d) Et_2_NH, THF, r.t., 87%. Boc-protected **61** was previously prepared by an alternative method; (e) HATU, Et_3_N, DMF; (f) Et_2_NH, THF, yields: 27–67% over two steps. *N*-Fmoc: protected amine.

Maderna et al. modified the Dov unit of Dol-10 with *α*,*α*-disubstituted amino acids and obtained a novel auristatin analog PF-06380101(**62**, Scheme 4). Compared with other existing auristatin analogs, **62** had outstanding curative effect and unique ADME characteristics in analyzing the proliferation of tumor cell. Firstly, the systemic clearance rate, distribution volume and elimination half-life (*t*_1/2_) of **62** were all in good condition, and no drug accumulation was observed during the interval of drug administration. Furthermore, compared with whole blood, **62** tendentiously distributed in human plasma as a substrate of P-glycoprotein(P-gp). Additionally, if **62** was administered in combination with clinical inhibitors and/or inducers of enzyme CYP3A4, drug-drug interactions might occur, since **62** was mainly metabolized by CYP3A4 [85].

**Figure 9:**
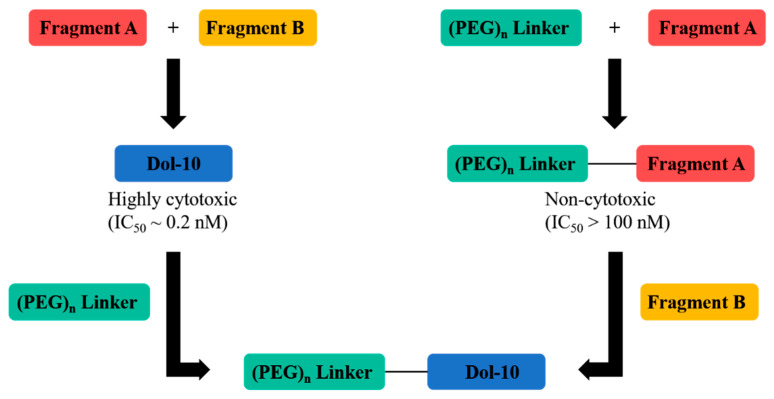
Synthetic plan of cytotoxic Dol-10 derivatives with hydrophilic drug linkers.

**Scheme 5:**
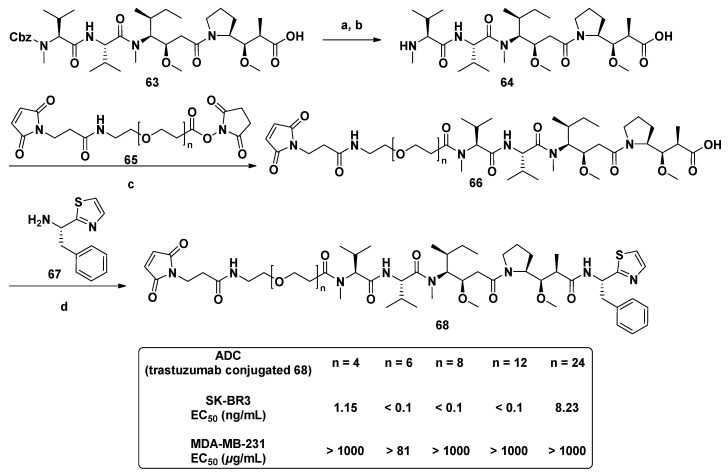
The synthesis of hydrophilic cytotoxic agent (**68**) containing PEG linker. Reagents and conditions: (a) HBr/AcOH; (b) Et_3_N, r.t.; (c) 1,4-dioxane, 80 °C; (d) DECP/DIPEA, r.t.

With the application of ADCs in cancer treatment, the coupling of Dol-10 derivatives with antibodies had been widely concerned. The hydrophilic Dov unit modified derivatives with maleimide group were synthesized through the polymerization and convergent synthesis (Figure 9). Discrete polyethylene glycols (PEG) with various chain lengths was introduced into the cytotoxic agent (**68**), which was used as drug linkers for synthesizing ADCs to increase hydrophilicity (Scheme 5). In cytotoxicity assay against SK-BR3 and MDA-MB-231 cells, the related ADC based on trastuzumab had excellent target specificity, and their cytotoxicity (EC_50_) was interfered with the length of the PEG linker. The solubility problem of ADCs related to hydrophobic anticancer drugs was also solved, and the natural highly cytotoxic peptide Dol-10 was avoided as starting material [99]. 

**Figure 10:**
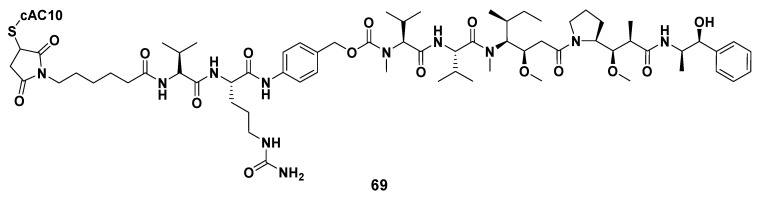
The chemical structure of ADC Adcetris^®^ (**69**).

Furthermore, some ADCs constructed by combining derivatives modified by Dov unit based on MMAE or MMAF with different antibodies have already entered clinical research or been authorized to market. MEDI-547, for example, was constructed by coupling the cytotoxic drug MMAF with the human anti-EphA2 monoclonal antibody (1C1). In in vitro experiments, MEDI-547 decreased the viability of Hec-1A and Ishikawa cells in endometrial carcinoma cell lines and accelerated their apoptosis. MEDI-547 also induced the degradation of EphA2, effectively inhibited the diffusion of tumor (the inhibition rate was about 85%) and diminished the metastasis rate of tumors. Because of these excellent performances, MEDI-547 was expected to treat patients with insufficient expression of EphA2 in malignant tumor cells. Nevertheless, in the open-label first-in-human examination, it was found that the safety characteristics of MEDI-547 did not allow further clinical research on patients with advanced solid tumors [100,101,102,103,104]. As the most successful representative of ADCs, Adcetris^®^ (also known as SGN-35, **69**, Figure 10), as a cAC10-Val-cit-MMAE conjugate constructed by conjugation of MMAE and anti-CD30 antibody (i.e., cAC10, a specific monoclonal antibody). Adcetris’s anti-CD30 antibody binds to CD30 antigen in Hodgkin disease cells and Le^Y^ antigen in cancer cells, while MMAE, the drug toxin, effectively inhibits mitosis by inhibiting tubulin polymerization. In 2011, Adcetris^®^ (Brentuximab vedotin for injection), jointly developed by Seattle Genetics and Takeda, was first marketed in the United States, and authorized to treat anaplastic large T-cell systemic malignant lymphoma and Hodgkin lymphoma [4,40,47,105,106,107,108,109]. At the beginning of August 2020, Takeda announced that Adcetris^®^ was officially listed in China, which brought new treatment options to patients in need. Up to now, Adcetris^®^ has benefited patients in more than 70 countries around the world, and it is also the most successful ADC in the field of hematology oncology in the world.

#### 3.1.6. Multiunit Modified Derivatives

**Scheme 6:**
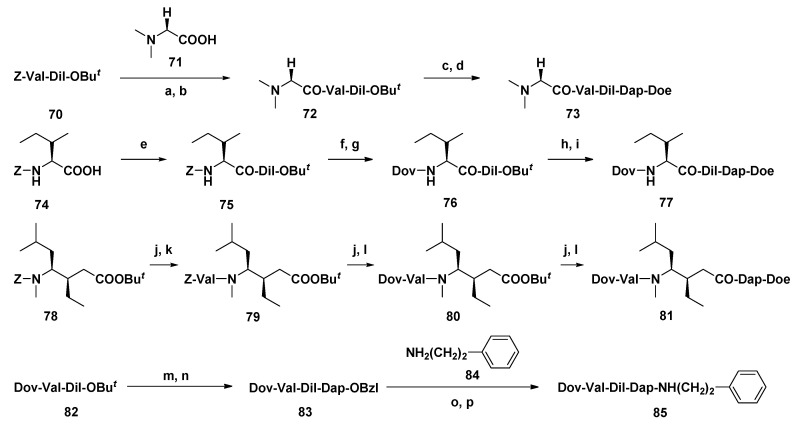
Synthesis of analogs modified at the Dov, Val, Dil, and Doe units. Reagents and conditions: (a) H_2_, Pd-C, *t*-BuOH/H_2_O; (b) DEPC, Et_3_N, DMF; (c) TFA, CH_2_Cl_2_; (d) H-Dap-Doe·HCl, DEPC, Et_3_N, DMF; (e) H-Dil-OBu*^t^*, DCC, CH_2_Cl_2_; (f) H_2_, Pd-C, *t*-BuOH/H_2_O; (g) Dov, DEPC, Et_3_N, DMF; (h) TFA, CH_2_Cl_2_; (i) H-Dap-Doe·HCl, DEPC, Et_3_N, DMF; (j) H_2_, Pd-C, *t*-BuOH/H_2_O; (k) *Z*-Val-OH, DCC, CH_2_Cl_2_; (l) Dov, DEPC, Et_3_N, DMF; (m) TFA, CH_2_Cl_2_; (n) H-Dap-OBzl·HCl, DEPC, Et_3_N, DMF; (o) H_2_, Pd-C, *t*-BuOH/H_2_O; (p) DEPC, Et_3_N, DMF.

By systematically modifying each subunit of Dol-10 in turn, a series of analogs with stronger antitumor activity than Dol-10 (Scheme 6) were developed by Miyazaki and co-workers. On the basis of P388 leukemia model in mice, the influences of subunit deletion and the change of functional group of each subunit were analyzed. The investigation had confirmed that derivatives **73**, **77**, **81**, and **85** modified by four different units had significant inhibitory effects on tumors in vivo (Table 4). Particularly, compound **85** lacking thiazole group of Doe unit was more effective than the parent compound **1** and was applied as a new candidate for antitumor agent [110].

The antiproliferative activities of Dol-10 (**1**), its homologues trisnordolastatin 10 (**86**), 9-*epi*-trisnordolastatin 10 (**87**), and their synthetic intermediates on L1210 mouse leukemia cells were evaluated by Shioiri et al. (Table 5). Compared with parent compound **1**, analogs **86** and **87**, intermediate tripeptide Boc-Dil-Dap-Doe (**89**), and dipeptide Boc-Dap-Doe (**90**) showed weaker antiproliferative activity, and only intermediate tetrapeptide Boc-Val-Dil-Dap-Doe (**88**) exhibited strong cytotoxicity similar to that of **1**. Therefore, it was speculated that the configuration at C-9 and *N*,*O*,*O*-trimethyl functional groups played a key role in the cytotoxicity of dolastatins [111]. 

#### 3.1.7. Cyclic Analogs

**Scheme 7:**
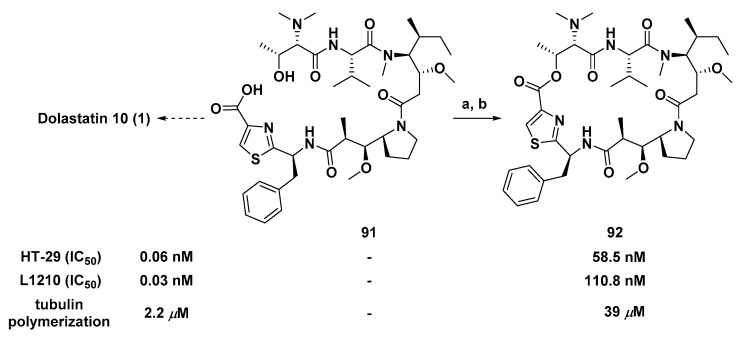
Synthesis of the cyclic analog (**92**) of Dol-10. Reagents and conditions: (a) IPCC/DMAP; (b) CH_2_Cl_2_, 3 h, r.t., 26%.

Different from common linear derivatives, the synthesis and activity of cyclic derivatives based on Dol-10 were rarely reported, which had aroused great interest of Poncet et al. Introducing an ester bond between the side chain of Dov residue and thiazole ring of Doe residue of compound **91** (Scheme 7), the modified linear precursor of Dol-10, to achieve the macrocyclic lactonization and obtain the cyclic analog (**92**) of the Dol-10. Compared with the parent compound **1**, the cytotoxicity of **92** against L1210 and HT-29 cell lines significantly decreased. Yet, it still had the submicromolar level inhibitory activity of microtubule polymerization in vitro (IC_50_ = 39 μM). The blocking of Dov residue modified in **91** resulted in a significant decrease in inhibitory activity, which indicated that Dov residue was crucial to the antiproliferative activity of Dol-10 [112].

### 3.2. Structure-Activity Relationship

**Figure 11:**
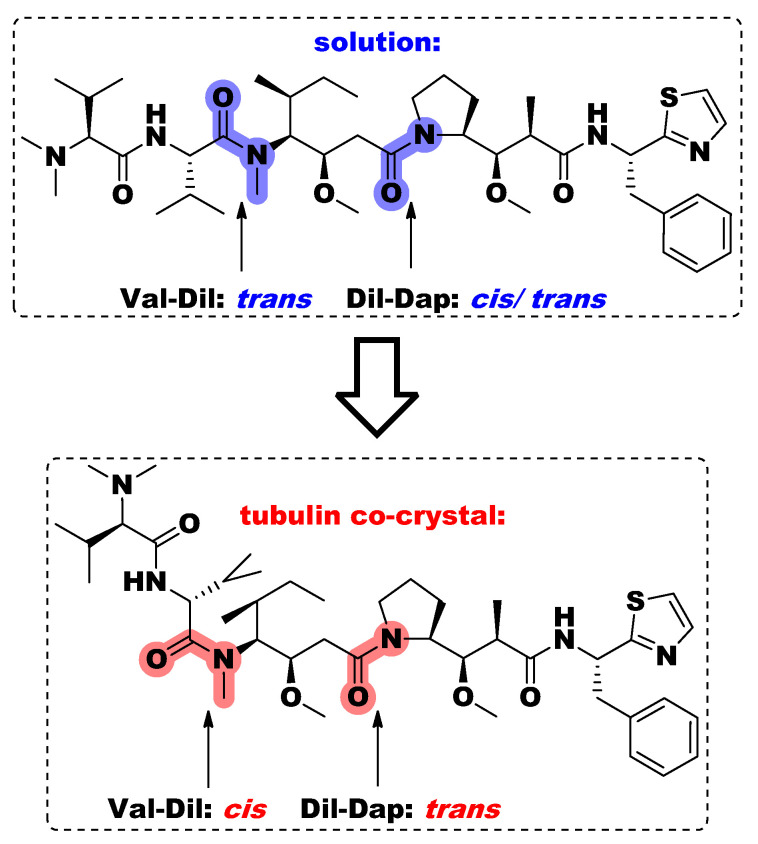
The geometric configurations of the Val-Dil amide bond and Dil-Dap amide bond of Dol-10 under different conditions.

Pentapeptide Dol-10 has nine asymmetric carbon atoms, of which five positions (C-9, 10, 18, 19, and 19a) possess the ability to obtain alternating configurations (Figure 1). The tubulin inhibitory activity of isomers was lost when C-18 and C-19 were modified, and only the reversal of C-19a configuration avoided the decrease of cytotoxicity. Nevertheless, there was no correlation between the effects of these isomers on microtubule assembly and cell growth [34]. Moreover, it was confirmed that both *N*-terminal tripeptide (lacking Dap and Doe), such as tripeptide A (**4**) and the *C*-terminal tetrapeptide (lacking Dov), inhibited tubulin polymerization but did not interrupt tubulin-ligand interaction. Only the antimitotic isomers with reversed configuration on C-6 or C-19a had the same activity as Dol-10. The stable binding (i.e., slow separation) of the peptide to tubulin preceded rapid binding, which significantly inhibited microtubule polymerization and interaction between other ligands and tubulin [33]. In solution, the geometric configurations of the Val-Dil amide bond and the Dil-Dap amide bond were *trans*- and *cis*-/*trans*-, respectively. According to speculation, the stability and configuration transition of Dil-Dap amide bond might be changed by the polarity of the solvent. However, configurations of the above two amide bonds showed *cis*- and *trans*- in the tubulin co-crystal, respectively (Figure 11). The phenomenon elaborated on the preferential binding mode of Dol-10 had guiding significance for the research of novel analogs with pre-oriented *cis*-Val-Dil amide bond and *trans*-Dil-Dap amide bond [83,85,113].

**Figure 12:**
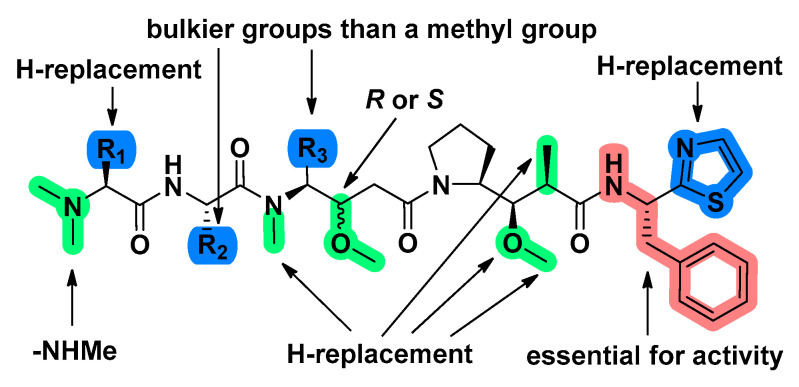
Structure-activity relationship of Dol-10 (**1**). The red marked part was an essential group for antitumor activity, and some modifications (marked with blue) had stronger activity than **1**, while other modifications (marked with green) possessed activity equivalent to **1**.

Some structural modifications are crucial to the antitumor activity of Dol-10 in vivo (Figure 12). For instance, substituting hydrogen for one *N*-methyl group in Dov unit, or *N*-methyl group in Dil unit, or 2-methyl group in Dap unit; replacing methoxy in Dap unit with hydrogen or hydroxyl group, and reversing the configuration of methoxy group in Dil unit failed to bring the significant difference to activity of Dol-10. However, the activity of modified products (such as **85**, Table 4) was significantly enhanced after deleting thiazole group in Doe unit. At the same time, if the thiazole group were deleted and the phenylethylamine moiety was simultaneously replaced by other aralkyl amide moieties, it would lead to the loss of cytotoxicity [84,110].

## 4. Synthetic Chemistry

**Figure 13:**
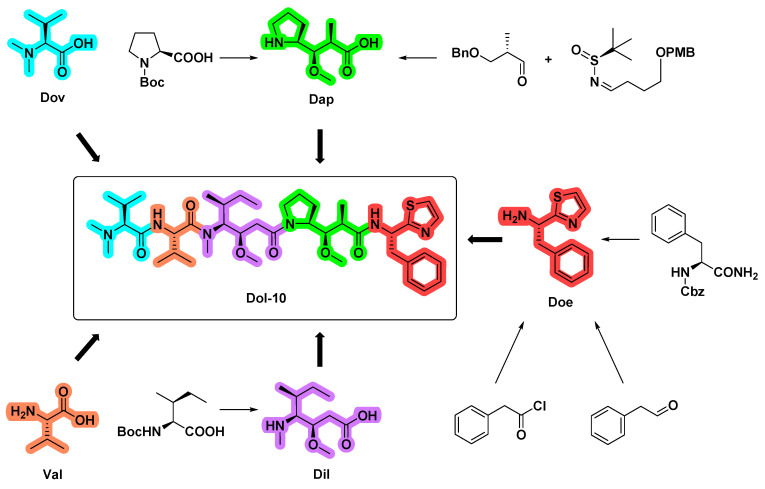
Overview of syntheses of Doe, Dap, Dil, and Dol-10.

The natural sources of the bioactive dolastatins, especially Dol-10, were rather limited (~1.0 mg/100 kg of collected organism from the sea hare *Dolabella Auricularia*, with a yield of 10^−6^~10^−7^%). Until the end of the 1980s, based on almost 20,000 times of separation and screening of two tons of raw materials by chromatography and mouse leukemia P388 model, Dol-10 was discovered by Pettit group for the first time [21,30,114]. Therefore, it is necessary to explore a reliable synthetic scheme for large-scale preparation of Dol-10, which will greatly accelerate the synthesis of potentially useful structures, extensive evaluation of biological characteristics and preclinical development. It is worth mentioning that the last three residues of Dol-10, Doe, Dap, and Dil, are particular to *Dolabella auricularia*, and their stereoselective synthesis is the key prerequisite for the total synthesis of the Dol-10 (**1**) and its analogs. In the activity evaluation of P388 lymphoblastic leukemia, the synthetic Dol-10 is equivalent to the corresponding natural product (ED_50_ = 1×10^−4^ μg/mL) [111]. According to the similarities and differences of synthetic strategies, the repeated or similar synthetic work is summarized (Figure 13), and more synthetic details and the conformational study of Dol-10 are introduced as follows.

### 4.1. Synthesis of the Doe Unit

**Scheme 8:**
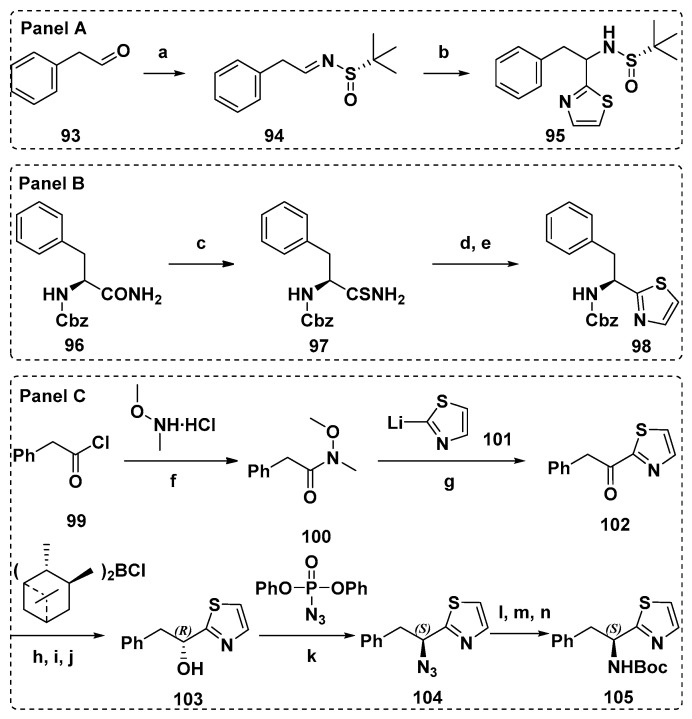
The preparation of the protected Doe fragments (**95**, **98**, and **105**). Reagents and conditions: (a) (*S*)-*tert*-Butanesulfinamide, CuSO_4_, PPTS, DCM, r.t., 36 h, 80%; (b) 2-bromothiazole, *n*-BuLi, toluene, −78 °C, 3 h, 53% (complete yield 80%); (c) Lawesson’s reagent, dioxane, 97%; (d) BrCH_2_CHO, K_2_CO_3_, DMF; (e) (CF_3_CO)_2_O, CH_2_Cl_2_, 66% in 2 steps; (f) pyridine, CH_2_Cl_2_, 0 °C, 2 h; r.t., 12 h, 77%; (g) THF, −78 °C, 30 min; 10 °C, 2 h, 56%; (h) Et_2_O, −10 °C; (i) NaOH, 0 °C; (j) H_2_O_2_, 10 °C to r.t., 81% yield, e.e. 92%, (60% yield, e.e. > 99%, after recrystallization); (k) PPh_3_, DEAD, THF, 0 °C to r.t., 48 h; (l) PPh_3_, 50 °C, 2 h; (m) NH_4_OH; (n) Boc_2_O, dioxane, 0 °C to r.t., 60 h, 44% (2 steps), e.e. > 99%.

A great deal of literatures have reported the synthesis of *C*-terminal Doe units in detail, which is mostly based on asymmetric addition reaction. compared with the method from Tomioka et al. [115], the asymmetric addition of chiral *N*-sulfinyl imine **94** (Scheme 8, Panel A) of Zhou et al. [98] was a simpler and more direct scheme to get the protected Doe fragment (**95**). Utilizing the *Z*-(*S*)-Phe-NH_2_ as raw material and improved Hantzsch method (Scheme 8, Panel B), *Z*-(*S*)-Doe (**98**) was prepared by Shioiri et al. Thioamide converted from *Z*-(*S*)-Phe-NH_2_ reacted with bromoacetaldehyde, and 4-hydroxythiazoline was dehydrated by trifluoroacetic anhydride to obtain the crude product **98**. Doe was obtained by repeated recrystallization from hexane-diethyl ether, with a yield of 64% and high optical purity (>97% e.e.) [116]. Later, in the optimization scheme of Burkhart et al., thiazole was synthesized by thio-Ugi reaction, and then Doe was directly synthesized. The improved scheme possessed the numerous advantages, such as simplicity, rapidity, high yield, and almost no racemization [117]. In 1992, Shioiri et al. also developed a brand-new scheme to obtain the key synthesis unit Doe [111], and the advancement of this scheme was verified by Mordant et al. (Scheme 8, Panel C). Phenylacetyl chloride **99** reacted with *N*-methoxy-*N*-methylamine hydrochloride to form amide **100**, which reacted with thiazolyllithium**101** produced benzyl thiazolyl ketone **102**, which was asymmetrically reduced by Brown’s reagent (Ipc_2_BCl) to afford (*R*)-alcohol **103**. With the help of Mitsunobu reaction, optically pure Boc-(*S*)-dorafenin**105** was finally obtained (44%, two steps) [118].

### 4.2. Synthesis of the Dap Unit

**Scheme 9:**
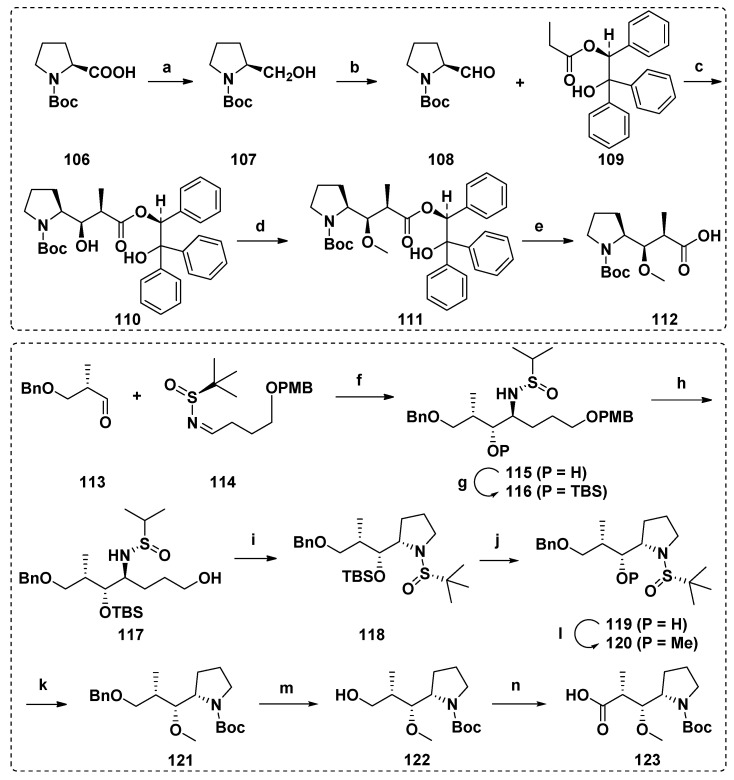
Synthesis of the Dap units (**112** and **123**). Reagents and conditions: (a) B_2_H_6_; (b) SO_3_·Py, Et_3_N, DMSO; (c) *i*Pr_2_NLi, MgBr_2_, Et_2_O; (d) (CH_3_)_3_OBF_4_, proton sponge; (e) H_2_, 10% Pd/C; (f) SmI_2_, *t*-BuOH, THF, −78 °C, 5 h, 75%; (g) TBSOTf, 2,6-lutidine, DCM, 0 °C to r.t., 4 h, 88%; (h) DDQ, DCM/H_2_O, 0 °C, 30 min, 68%; (i) (1) MsCl, TEA, DCM, 0 °C, 15 min; (2) *t*-BuOK, THF, 0 °C, 15 min, for two steps 73%; (j) TBAF, THF, 0 °C to r.t., 4 h, 61%; (k) LiHMDS, HMPA, THF, −78 °C, 30 min, and then MeOTf, −15 °C, 15 min, 95%; (l) (1) HCl/dioxane, MeOH, 0 °C, 30 min; (2) Boc_2_O, TEA, DCM, r.t., 12 h, for two steps 79%; (m) Pd/C, H_2_, MeOH, 5 h, 72%; (n) (1) DMP, DCM, r.t., 30 min; (2) NaH_2_PO_4_·2H_2_O, NaClO_2_, 2-methyl-2-butene/*t*-BuOH, r.t., 8 h, for two steps 75%.

There are many reports about the synthesis of Dap unit, and the typical one is the *N*-*tert*-butoxy carbonyl derivative **112** of Dap synthesized by Pettit et al. Aldehyde **108** derived from (*S*)-proline and chiral propionate **109** were assembled through Evans aldol condensation to obtain related diastereomers of Dap. Methyl ether **112** was then produced by hydrogenolysis-based methylation and cleavage of chiral-directed ester groups (Scheme 9) [71,111,116,119]. In addition, there are many other reports on the construction of Dap unit, including crotylation of natural amino acid *N*-Boc-*L*-prolinal [97], asymmetric hydrogenation of *β*-keto esters derived from (*S*)-Boc-proline under the catalysis of ruthenium-SYNPHOS complex [118], or aldehyde cross-coupling with (*S*)-*N*-*tert*-butyrylimine under SmI_2_ induction (Scheme 9) [98].

### 4.3. Synthesis of the Dil Unit

**Scheme 10:**
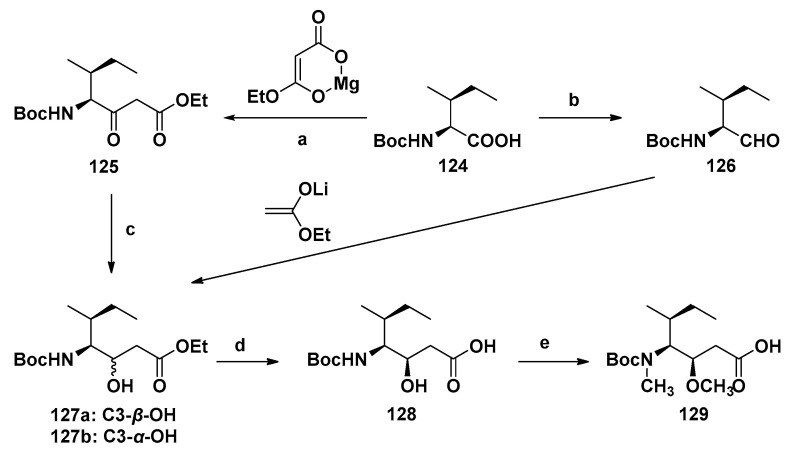
Synthesis of the Dil unit (**129**). Reagents and conditions: (a) CDI (carbonyl dimidazole), THF; (b) (1) CH_3_I, KHCO_3_, (2) LiCl, NaBH_4_, (3) DMSO, Py·SO_3_; (c) NaBH_4_; (d) NaOH; (e) (1) NaH, (2) CH_3_I.

The complete preparation scheme of the Dil unit was first developed by the Hamada group [111,116]. With Boc-(*S*)-isoleucine (**124**) or Boc-(*S*)-isoleucinal (**126**) as raw materials, the chiral center needed was established in a stereoselective manner, and then the methylation of nitrogen and oxygen realized the efficient synthesis of Dil unit (**129**, Scheme 10). Firstly, **124** was converted into the corresponding imidazoline, which was treated with magnesium enolate of malonate half ester to obtain *β*-keto ester **125**, subsequently reduced with sodium borohydride to get a mixture of hydroxyl esters **127a** and **127b** with a ratio of 91:9. The reaction of Boc-(*S*)-isoleucine (**126**) with lithium enol, an ideal alternative method, obtained **127a** and **127b** with a ratio of 38:62. Alkaline hydrolysis of the separated hydroxyl ester **127a** produced carboxylic acid **128**, which was finally treated to yield the target product Dil unit **129**. This synthetic scheme was mature and efficient, and it has been widely used in later related research [97,98,118,119].

### 4.4. Synthesis of Dolastatin 10

**Scheme 11:**
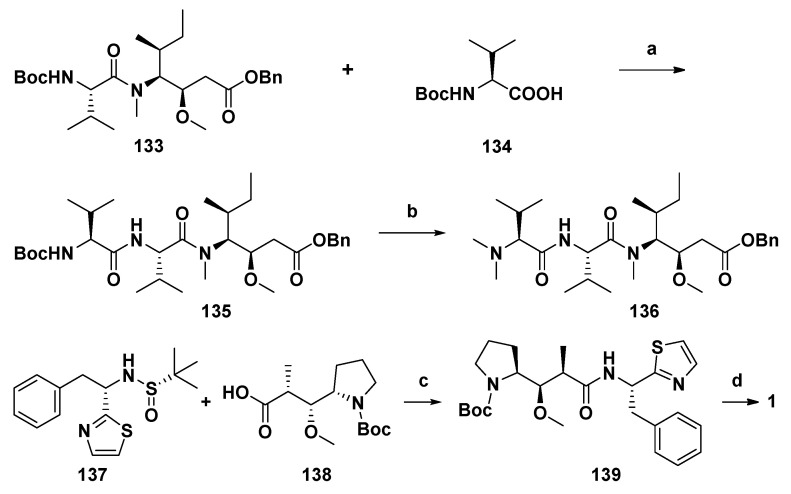
The asymmetric synthesis of Dol-10 (**1**). Reagents and conditions: Please refer to Reference [98] for the synthesis of **133**. (a) (1) TFA, DCM, 0 °C, 2 h; (2) HATU, HOAt, DIPEA, DCM, r.t., overnight, for two steps 85%; (b) (1) TFA, DCM, r.t., 2 h; (2) 40% HCHO, Na(BH_3_)CN, CH_3_CN, r.t., 18 h, for two steps 82%; (c) (1) HCl/dioxane, MeOH, 0 °C, 30 min; (2) HATU, HOAt, DIPEA, DCM, r.t., overnight, for two steps 80%; (d) (1) TFA, DCM, 0 °C, 2 h; (2) **136**, Pd/C, H_2_, MeOH, 2 h; (3) HATU, HOAt, DIPEA, DCM, r.t., 24 h, for three steps 60%.

**Scheme 12:**
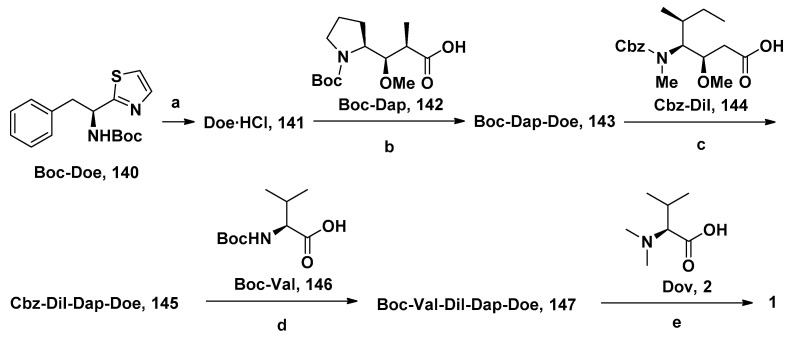
The linear synthesis pattern of Dol-10 (**1**) starting from *C*-terminal. Reagents and conditions: (a) 2.4 N HCl/dioxane, r.t., 80 min, 93%; (b) DEPC, NEt_3_/DME, 4 °C, 14 h, 78%; (c) 2.4 N HCl/dioxane, r.t., 1 h, DEPC, NEt_3_/DME, 0 °C, 3 h, 70%; (d) 5% HBr/AcOH, r.t., 2 h, BopCl, NEt_3_/CH_2_Cl_2_, 4 °C, 7 d, 75%; (e) 1.2 N HCl/dioxane, r.t., 1 h, DEPC, NEt_3_/DME, 4 °C, 12 h, 75%.

According to most reports, two synthetic schemes of Dol-10 based on Evans aldol method are summarized. In the asymmetric synthesis scheme of Pettit and Zhou et al. (Scheme 11) [98,120], the *C*-terminal subunit (*S*)-Doe and remaining four amino acid residues were gradually assembled into compound **1** with high stereoselectivity, which were more convenient, effective and acceptable than most linear synthesis patterns starting from *C*-terminal (Scheme 12) [111,116,118,119].

### 4.5. Conformational Study

The basic conformational characteristics of Dol-10, the effect of terminal residues on the whole conformation and interaction of peptides were described via nuclear magnetic resonance (NMR), molecular mechanics (MM) and molecular dynamics (MD) calculation techniques [113,121,122,123].

**Figure 14:**
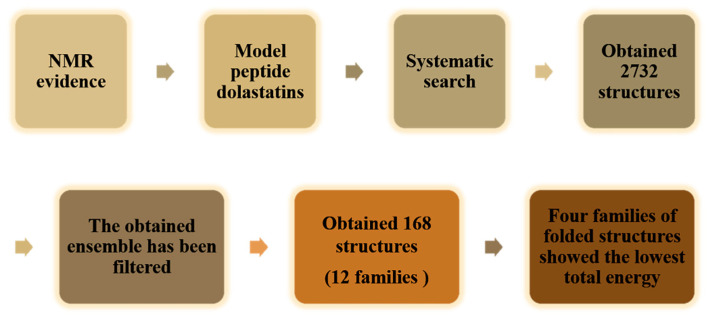
Outline of the procedures for the conformational search performed.

The ^1^H NMR spectra and ^1^H-^13^C hetero-correlated spectra were analyzed in CD_2_Cl_2_ solution, and the conformational energy minimization was systematically studied under limited experimental conditions. According to the NMR data, it was observed that the *a*CH (25) proton of Dov residue had a huge shielding effect, which indicated that there was an interaction between *N*-terminal and aromatic *C*-terminal of the peptide. Therefore, it was speculated that this linear dolastatin molecule might have a ring-like conformation. However, the conformational theory analysis denied the possibility of binding between molecules from head to tail, verified the NMR hypothesis of a folded peptide-like molecule, and speculated a series of possible conformations based on the consistent experimental data (Figure 14) [121]. Molecular mechanics (MM) analysis confirmed that pentapeptide had a comparatively rigid molecular system. Molecular dynamics (MD) simulation of molecular conformation had reached an almost stable state in the gas phase without large degree of conformational freedom. The conformation with the least energy had a pocket-like shape with all groups facing out of the structural framework. In addition, the conformational behavior of Dol-10 mainly depended on the hydrophobicity of its residues. On the *C*-terminal residue, even slight structural modifications might change the functional position of interaction with biological partners [123]. The model studies indicated that the terminal residues Dov and Doe had certain mobility, while the internal residues Val and Dap had almost no change in *cis*- and *trans*- structures. On the contrary, due to the *cis*-*trans* isomerization of the C15-C16 amide bond, the central residue Dil changed significantly [113].

To sum up, this work was helpful to understand the structural characteristics of Dol-10 that played a role in microtubule polymerization and mitosis. The folding structure and high flexibility of Dol-10 together realized its excellent biological activity.

## 5. Conclusions

Marine bioactive polypeptide Dol-10 has been investigated for more than 30 years, from its first discovery to the approval of the related drug for marketing, which brings a bright prospect for tumor treatment and is still one of the most active antitumor compounds found so far. Dol-10 and its derivatives can effectively inhibit the growth of tumor cells in vitro, but their clinical efficacy as a single drug in phase II clinical trials of solid tumors is frustrating due to adverse effects. For example, the most common peripheral neuropathy had brought a great blow to the clinical research of the marine peptide. Later, chemical researchers coupled monoclonal antibodies with Dol-10 derivatives by ADCs technology, and utilized the specificity of antibodies to transport drug molecules to target tissues for their functions. As a result, the systemic toxic and side effects of drugs were reduced, and the treatment window of drugs was improved. Adcetris^®^, as a typical representative of listed ADCs, has successfully overcome the obstacles in clinical application of Dol-10 and greatly promoted the research progress of ADCs. Moreover, in the process of systematical antitumor study of Dol-10, it is accidentally found that this antitumor peptide also has potent antifungal [124], antibacterial [86], and anti-*Plasmodium falciparum* [125] biological activities, which points out a new direction for the further development of Dol-10. At present, many excellent studies about Dol-10 have been published, but the related antitumor research based on Dol-10 still faces challenges and opportunities. Reducing the toxicity of Dol-10 in vivo and in clinical research by structural modifications and/or combining with newly emerging medical technology, so that it can be better applied to the treatment of tumors. Considering most reported structural modifications of *N*- and *C*-terminal subunits, it can be predicted that more Dol-10 analogs based on modified central residues Dil, Dap, and Doe will appear soon. And there is no doubt that selecting appropriate monoclonal antibodies to couple with Dol-10 or its derivatives to prepare ADCs into the clinic is still one of the main research directions of Dol-10 in the future.

## Figures and Tables

**Table 1:** Therapeutic use and evaluation of Dol-10 in various antitumor research.

Therapeutic Use	Development State	Evaluation Outcome *	Reference
Anti-lymphoma	Preclinical	++	[36,49,50,51]
Anti-lung cancer	Preclinical	++	[37]
Anti-ovarian carcinoma	Phase II	+	[48]
Anti-prostate cancer	Preclinical, phase II	++, + (respectively)	[36,52]
Anti-soft tissue sarcoma	Phase II	-	[53]
Anti-breast cancer	Phase II	-	[54]
Anti-hepatobiliary pancreatic carcinoma	Phase II	-	[55]

*-, almost no objective curative effect and toxic symptoms were observed; +, positive; ++, strongly positive.

**Table 2:**
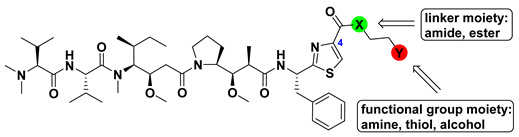
Structure-activity relationships for Dol-10 derivatives with carbonyl functional group at the C-4 of thiazole of Doe unit.

Compound	-X-	-Y	IC_50_ (nM)		
			SKOV-3	A549	L1210
MMAE (**7**)	-	-	0.66	1.3	2.1
**18**	-O-	-H	0.024	-	-
**20**	-NH-	-H	0.55	0.74	6.5
**21a**	-NH-	-NH_2_	>10	>10	>50
**21b**	-NH-	-NHMe	>10	9.9	27
**21c**	-NH-	-OH	4.7	1.7	28
**21d**	-NH-	-SH	0.44	1.9	31
**21e**	-NH-	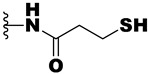	>10	>10	>50
**21f**	-NH-	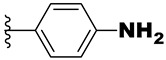	0.27	1.1	4.1
**21g**	-NH-	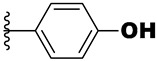	0.37	1.3	5.6
**22a**	-NMe-	-NH_2_	0.56	0.81	12
**22b**	-NMe-	-NHMe	0.19	1.9	14
**22c**	-NMe-	-OH	1.5	1.6	8.5
**22d**	-NMe-	-SH	1.1	1.2	7.0
**23a**	-O-	-NH_2_	0.85	0.83	29
**23b**	-O-	-OH	0.086	1.8	4.7
**23c**	-O-	-SH	0.13	6.9	14

**Table 3:** The growth inhibition of **24**, **25**, **26**, and **27** in murine and human cancer cell lines (ED_50_ (μg/mL) and GI_50_ (nM)) ^a^.

Compound	Cell Line ^b^						
	P388	NCI-H460	KM20L2	DU-145	BXPC-3	MCF-7	SF-268
**24**	<0.001 (<1.2)	0.00088 (1.05)	0.00061 (0.72)	0.00054 (0.64)	0.046 (54.6)	0.00068 (0.81)	0.00125 (1.48)
**25**	0.031 (42.8)	0.0160 (22.1)	0.0077 (10.6)	0.023 (31.8)	0.029 (40.1)	0.0046 (6.35)	0.029 (40.1)
**26**	0.0026 (3.59)	0.00036 (0.50)	0.00025 (0.35)	0.00030 (0.41)	0.00031 (0.43)	0.00014 (0.19)	0.00016 (0.22)
**27**	-	-	-	-	-	-	-
	(-)	(198.0)	(35.82)	(12.26)	(386.5)	(35.82)	(30.17)

^a^ The nanomolar values in brackets are the cytotoxicity concentrations. ^b^ The sequence of tumor cell lines: murine lymphocytic leukemia (P388), lung (NCI-H460), colon (KM20L2), prostate (DU-145), pancreas (BXPC-3), breast (MCF-7), and CNS (SF-268).

**Table 4:** Antitumor activity of multiunit modified Dol-10 derivatives. Position: Dov^1^-Val^2^-Dil^3^-Dap^4^-Doe^5^.

Compound	Modified Position	ILS_max_ *	Optimal Dose (mg/kg per inj.)
**1**		50	0.05
**73**	1	94	0.5
**77**	2	80	0.5
**81**	3	74	2.0
**85**	5	83	0.5

* Maximal increase in life span (please refer to Reference [110]).

**Table 5:**
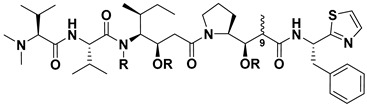
Chemical structures and cytotoxicity of Dol-10 (**1**) and its derivatives **86**–**90**.

	Compound	R	C_9_-CH_3_	IC_50_ (μg/mL)
**1**	Dol-10	CH_3_	*β*	2.95 × 10^−4^
**86**	trisnordolastatin 10	H	*β*	1.0 × 10^−1^
**87**	9-*epi*-trisnordolastatin 10	H	*α*	50
**88**	Boc-Val-Dil-Dap-Doe			4.0 × 10^−3^
**89**	Boc-Dil-Dap-Doe			11
**90**	Boc-Dap-Doe	CH_3_	*β*	>100
